# Screening and preliminary identification of long non-coding RNAs critical for osteogenic differentiation of human umbilical cord mesenchymal stem cells

**DOI:** 10.1080/21655979.2022.2044274

**Published:** 2022-03-07

**Authors:** Xiao Zheng, Shuaiqi Gan, Cheng Su, Zheng Zheng, Yihan Liao, Jingjing Shao, Zhimin Zhu, Wenchuan Chen

**Affiliations:** a State Key Laboratory of Oral Diseases and National Clinical Research Center for Oral Diseases, West China Hospital of Stomatology, Sichuan University, Chengdu, Sichuan, China; b Department of Oral Prosthodontics, West China Hospital of Stomatology, Sichuan University, Chengdu, Sichuan, China; c Department of Pediatric Dentistry, Shenzhen Stomatology Hospital (Pingshan), Southern Medical University, Shenzhen, Guangdong, China

**Keywords:** Human umbilical cord mesenchymal stem cell, long non-coding RNAs, osteogenic differentiation, gene expression

## Abstract

Human umbilical cord mesenchymal stem cells (hUCMSCs) are attractive therapeutic cells for tissue engineering to treat bone defects. However, how the cells can differentiate into bone remains unclear. Long non-coding RNAs (lncRNAs) are non-coding RNAs that participate in many biological processes, including stem cell differentiation. In this study, we investigated the profiles and functions of lncRNAs in the osteogenic differentiation of hUCMSCs. We identified 343 lncRNAs differentially expressed during osteogenic differentiation, of which 115 were upregulated and 228 were downregulated. We further analyzed these lncRNAs using bioinformatic analyses, including Gene Ontology (GO) and Kyoto Encyclopedia of Genes and Genomes (KEGG) pathway analysis. GO and KEGG pathway analysis showed that ‘intracellular part’ and ‘Phosphatidylinositol signaling system’ were the most correlated molecular function and pathway, respectively. We selected the top 10 upregulated lncRNAs to construct six competing endogenous RNA networks. We validated the impact of the lncRNA H19 on osteogenic differentiation by overexpressing it in hUCMSCs. Overall, our results pave the way to detailed studies of the molecular mechanisms of hUCMSC osteogenic differentiation, and they provide a new theoretical basis to guide the therapeutic application of hUCMSCs.

## Graphical abstract

**Figure:**
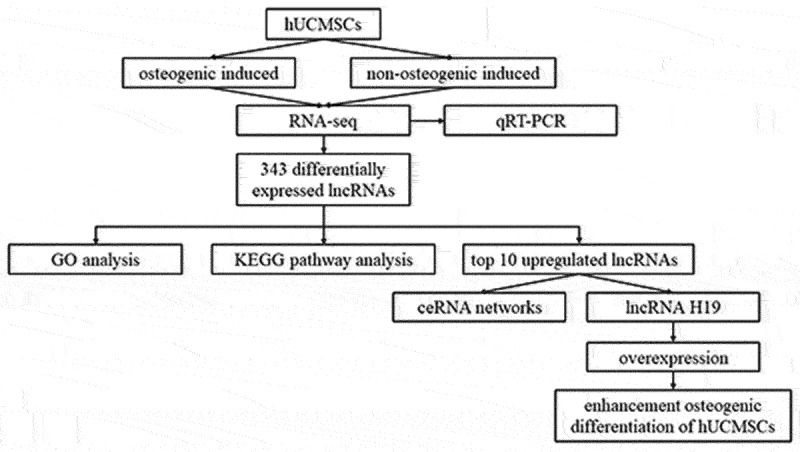

## Introduction

Bone defects caused by tumor resection, infection or trauma are common [1]. Cell-based tissue engineering using mesenchymal stem cells (MSCs) has emerged as a new approach for bone repair and reconstruction [2]. MSCs are cells capable of self-regenerating and differentiating along multiple lineages. Bone marrow mesenchymal stem cells (BMSCs) are one of the most studied types of MSCs and have shown promising clinical results for regenerative bone therapy [3]. However, human BMSCs (hBMSCs) are not always applicable in the clinical setting because of several drawbacks, including the need for invasive harvesting [4], slow proliferation *in vitro* [5], and insufficient quantity and quality in older and diseased individuals [6]. A promising alternative is human umbilical cord mesenchymal stem cells (hUCMSCs) [7], which can easily be obtained from the umbilical cord after delivery [8] and which proliferate rapidly *in vitro* and are less immunogenic than BMSCs [9,10]. In addition, hUCMSCs seem to have the similar osteogenic ability as BMSCs [11]. To exploit hUCMSCs for bone therapy, how they differentiate into bone tissue must be clarified.

Many transcriptional pathways and post-transcriptional pathways regulate MSC differentiation [12]. Extensive studies have revealed that long non-coding RNAs (lncRNAs) regulate the differentiation of MSCs [13]. Non-coding RNAs do not code for proteins but play an important role in epigenetic control; they are divided into lncRNAs, which are longer than 200 nucleotides, and microRNAs (miRNAs), which are 18–22 nucleotides long [14]. While miRNAs inhibit translation of mRNAs or trigger their degradation by binding to the 3’ untranslated region [15], lncRNAs can interact with DNA, RNA, and protein to regulate gene expression [^16–18^]. Some lncRNAs affect osteogenesis of BMSCs by regulating Wnt-β-catenin signaling [19] and TGF-β1/Smad3/HDAC signaling [20], as well as by interacting with the miRNAs miR-188 [21] and miR-138 [22]. Besides, several lncRNAs are crucial regulators of MSC osteogenic differentiation [20^,23–28^]. In contrast, we are unaware of reports linking lncRNAs to osteogenesis of hUCMSCs.

Recent advances in RNA sequencing and bioinformatics allow detailed analysis of non-coding RNAs, making it possible to identify transmitters and receivers in RNA regulatory networks [29]. Therefore, in the present study, we investigated the expression and potential functions of lncRNAs, including lncRNA H19, in the osteogenic differentiation of hUCMSCs. Our findings may help clarify the mechanisms of osteogenic differentiation of hUCMSCs, facilitating the exploitation of hUCMSCs for regenerative bone therapy.

## Materials and methods

### Cell culture

hUCMSCs were purchased from Sichuan Neo-life Stem Cell Biotech (Chengdu, China) and cultured in low-glucose Dulbecco’s Modified Eagle Medium (GIBCO, USA) containing 10% fetal bovine serum (GIBCO, USA) and 1% (v/v) penicillin/streptomycin (Hyclone, USA) (hUCMSC growth medium) [4]. We cultured cells in a humidified atmosphere containing 5% CO_2_ at 37°C. Cells were detached using 0.25% trypsin (Hyclone, USA) and passaged.

### Osteogenic differentiation of hUCMSCs

Fourth-passage hUCMSCs were used for osteogenic induction. hUCMSCs with 70–80% confluence were cultured in the osteogenic medium, which contains hUCMSC growth medium plus 10 mM *β*-glycerol phosphate, 50 μM ascorbic acid, and 100 nM dexamethasone (all from Sigma-Aldrich, USA) [30]. The medium was replaced every two days.

### Alkaline phosphatase (ALP) staining

We seeded hUCMSCs in 6-well plates at a density of 200,000 cells per well and cultured them in osteogenic medium. We replaced the medium every two days. On day 7, ALP staining was performed using a BCIP/NBT Alkaline Phosphatase Color Development Kit (Beyotime, China) following the instructions from the manufacturer [31]. Briefly, we fixed the cells in 4% paraformaldehyde (Solarbio, China) for 30 min at room temperature (RT), washed the cells three times with PBS (GIBCO, USA), and stained the cells with NBT/BCIP solution for 24 h at RT. Then we removed the staining solution, washed the cells three times with PBS, and observed them under an optical microscope (OLYMPUS, Japan). The ALP staining images were semi-quantified using ImageJ (version 1.6.0, Wayne Rasband, National Institute of Health, USA) as described previously [32,33].

### Alizarin red staining

We cultured cells the same as for ALP staining and on day 21, we stained the cells with Alizarin red [34]. Briefly, we fixed the cells in 4% paraformaldehyde for 30 min at RT, washed them three times with distilled water, and stained them with 1% Alizarin red staining solution (Solarbio, China) for 30 min at RT. Then we removed the staining solution, washed the cells three times with distilled water, and observed them under an optical microscope (OLYMPUS, Japan). The semi-quantitative analysis of Alizarin red staining was performed using ImageJ (version 1.6.0, Wayne Rasband, National Institute of Health, USA) as described previously [32,33].

### Total RNA isolation and quantitation

We extracted total RNA from hUCMSCs using TRIzol (Invitrogen, USA) following the instructions from the manufacturer [35]. Briefly, we lysed cells in 6-well plates with 1 ml TRIzol per well for 5 min at RT. Then we transferred the lysed cells into an Eppendorf, added 0.2 mL of chloroform, and thoroughly mixed by shaking for 15 sec. After 3 minutes’ incubation, we centrifuged the mixture for 15 min at 12,000 g at 4°C. Then the aqueous phase containing the RNA was transferred to a new Eppendorf, 0.5 mL of isopropanol was added, and the tube was incubated for 10 min at 4°C, then centrifuged for 10 min at 12,000 g at 4°C. The pellet containing the RNA was resuspended with 1 ml of 70% RNase-free ethanol and centrifuged at 8,000 rpm for 30 sec. The RNA pellet was air-dried for 10 min and resuspended in 20 µL of RNase-free water. RNA quality and quantity were evaluated using a NanoDrop 2000 spectrophotometer (Thermo Scientific, USA). Only RNA samples with an absorbance ratio 260/280 > 1.8 were analyzed further. RNA integrity and purity were assessed using 1% agarose gel electrophoresis. Quality-checked RNA was stored at −80°C.

### Quantitative real-time PCR (qRT-PCR)

qRT-PCR was performed to verify the effectiveness of osteogenic differentiation of hUCMSCs, to validate RNA sequencing, and to confirm lncRNA H19 overexpression. In each case, quality-checked total RNA was reverse-transcribed into cDNA using a RevertAid First Strand cDNA Synthesis Kit (Thermo Scientific), which then served as template to amplify target genes in a Lightcycler96 System (Roche, USA) using Hieff^TM^ qPCR SYBR® Green Master Mix (YEASEN, China) [36]. Primer sequences for the various target genes are listed in Tables 1 and 2

**Table 1.** Primers of internal control and osteogenesis-related markers

Gene	Forward primer sequence(5’-3’)	Reverse primer sequence(5’-3’)	Accession number
GAPDH	TGCACCACCAACTGCTTAGC	GGCATGGACTGTGGTCATGAG	NM_001256799.3
ALP	GACCTCCTCGGAAGACACTC	TGAAGGGCTTCTTGTCTGT	NM_001127501.4
RUNX2	TCCACACCATTAGGGACCATC	TGCTAATGCTTCGTGTTTCCA	NM_001015051.4
OPG	CGCTCGTGTTTCTGGACATCT	CACACGGTCTTCCACTTTGC	NM_002546.4
OPN	TCTGGGAGGGCTTGGTTGTC	TTTCCTTGGTCGGCGTTTG	NM_001308174.2
OCN	CCCAGGCGCTACCTGTATCAA	GGTCAGCCAACTCGTCACAGTC	NM_000711.1

**Table 2.** Primers used for validating sequencing results and confirming lncRNA H19 overexpression

Gene	Forward primer sequence(5’-3’)	Reverse primer sequence(5’-3’)
ENST00000414790	CTTTCATGTTGTGGGTTCTGG	CGGGTCTGTTTCTTTACTTCC
ENST00000580476	CTGAGGTCGGCGGATCGT	CAACACGGGGAGTTTGACCT
ENST00000577988	TTTTCGCCTCCTGTTTCAGC	ACAGAACAAGAGACCCGGAG
ENST00000363359	TGTAGAGCACCGAAAACCCC	ACTCAGACCGCGTTCTCTC
ENST00000584923	GTTTTCTCGGGGTGGCTTTT	ACAGAACAAGAGACCCGGAG
NR_027405	CTAGGTCAGGTTGGAGTGCA	CCCACTCTTCTACCTCCTGC
ENST00000428008	AGTTCCCGTTTTGTGTGTGG	TCTACTTCCAACACCCGCAT
ENST00000448718	GAGCAAGCCTAACTCAAGCC	ACACAGTGTAGTCAAGCCGA
ENST00000483140	CCTCAATCACCCAGGCCTAA	CTGTGCCTTTGGAAGCTGAG
NR_109779	CCTGCCGGATTGCTTTTCTT	TGCCCCTCATCACCAAATCT
H19	CTTTCATGTTGTGGGTTCTGG	CGGGTCTGTTTCTTTACTTCC

. Amplification was performed with the following cycling conditions: 95°C for 5 min, then 40 cycles of 95°C for 10 sec, 60°C for 20 sec, and 72°C for 20 sec. We quantified levels of target lncRNAs using the 2^−ΔΔCq^ method relative to the level of mRNA encoding glyceraldehyde-3-phosphate dehydrogenase (GAPDH).

### RNA sequencing

The isolated total RNA from hUCMSCs on day 7 was sequenced by CloudSeq Biotech (Shanghai, China). Briefly, we removed ribosomal RNA using the Ribo-Zero rRNA Removal Kits (Illumina, USA), then used the resulting RNA samples to construct RNA libraries with the TruSeq Stranded Total RNA Library Prep Kit (Illumina, USA). Libraries were assessed quantitatively and qualitatively using the BioAnalyzer 2100 system (Agilent Technologies, USA), then they were sequenced using an Illumina HiSeq 4000 sequencer (LC Biotech, China). Paired-end reads from the sequencer were checked for quality using Q30. Raw reads were subjected to 3’ adaptor-trimming, and low-quality reads were removed using Cutadapt software (version 1.9.3) [37]. The resulting high-quality trimmed reads were analyzed for lncRNAs by first mapping them to the human reference genome (UCSC hg19) using Hisat2 software (version 2.0.4) [38], and then assembling and annotating transcripts using Cufflinks (version 2.2.1) [39] based on the Ensembl gtf gene annotation file. Expression of lncRNAs was calculated in terms of fragments per kilobase of exon per million fragments mapped (FPKM).

### Functional enrichment analysis

The potential functions of lncRNAs were explored in terms of Gene Ontology functions (www.geneontology.org) and Kyoto Encyclopedia of Genes and Genomes (KEGG) pathways (www.kegg.jp), based on the functions and pathways of coding genes nearest to the lncRNAs [40]. Results were considered significant if associated with *P* < 0.05.

### Construction of the competing endogenous RNA (ceRNA) network

The lncRNA-miRNA-mRNA-associated ceRNA network depicts which lncRNAs and mRNAs compete for the same pool of miRNAs [41]. To construct this ceRNA network, we combined the lncRNA-miRNA network with the miRNA-mRNA network. First, differentially expressed lncRNAs were named as in the miRcode database, then names of miRNAs were retrieved and the lncRNA-miRNA network was predicted [42]. Next, we generated the miRNA-mRNA network using Targetscan [43], miRTarbase [44], and miRDB [45]. Finally, the ceRNA network was constructed using Cytoscape (version 3.8.2).

### Adenovirus construction and infection of hUCMSCs

Recombinant adenoviruses were constructed by Hanbio (Shanghai, China) and used to infect hUCMSCs as described [36] at a multiplicity of infection of 10 for 8 h. Cells were infected with viruses encoded the lncRNA H19 or, as a control, green fluorescent protein (GFP). Uninfected cells were used as nonspecific control cells. The cells were incubated in osteogenic medium. On day 3, we extracted total RNA from the cells and performed qRT-PCR to determine the expression of lncRNA H19. On day 7, ALP staining was performed, and total RNA was again extracted and analyzed for expression of the osteogenesis-related genes encoding ALP, runt-related transcription factor 2 (RUNX2), osteocalcin (OCN), and osteoprotegerin (OPG).

### Statistical analysis

All experiments were performed at least three times. All statistical analyses were performed using SPSS version 21.0 (SPSS, Chicago, IL, USA). Data were expressed as mean ± standard deviation, and differences between two groups were assessed for significance using the independent-samples t test and one-way ANOVA. Differences associated with *P* < 0.05 were considered significant.

## Results

In our study, we used RNA sequencing to identify lncRNAs differentially expressed during the osteogenic differentiation of hUCMSCs. After validating the sequencing results using qRT-PCR, we analyzed the potential functions of the differentially expressed lncRNAs based on GO terms and KEGG pathways. Then we used the top 10 upregulated lncRNAs to construct ceRNA networks, and we validated the impact of lncRNA H19 on osteogenic differentiation by overexpressing it in hUCMSCs.

### Osteogenic differentiation of hUCMSCs

**Figure 1.**
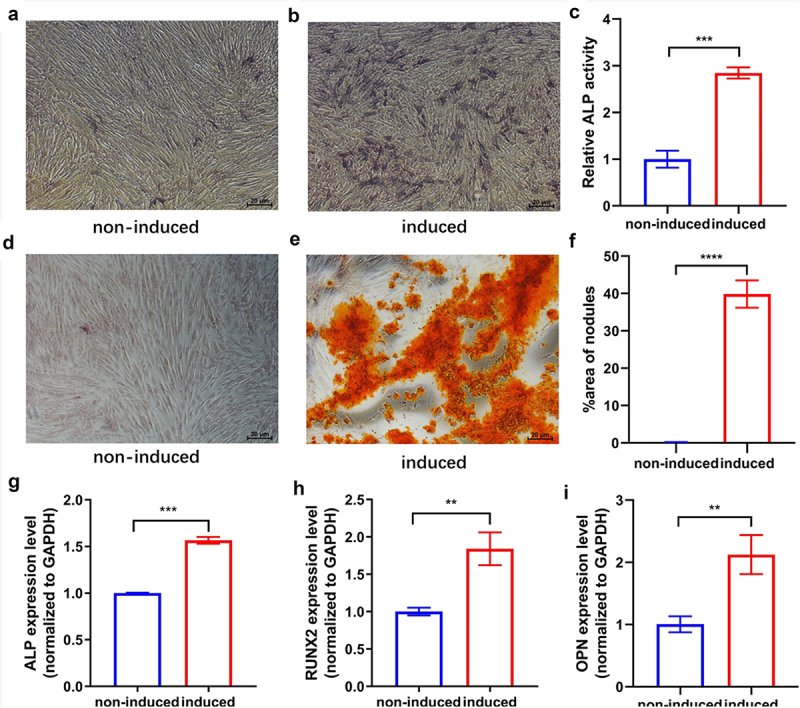
Osteogenic differentiation of hUCMSCs. Cultures were either induced to undergo osteogenic differentiation (‘induced’) or not (‘non-induced’). (a-b) ALP staining on day 7. (c) Semi-quantitative analysis of ALP activity on day 7. (d-e) Alizarin red staining on day 21. (f) Semi-quantitative analysis of calcium nodule deposition on day 21. (g-i) Expression levels of the osteogenesis-related markers ALP, RUNX2, and OPN on day 7. **P < 0.01, ***P < 0.001, ****P < 0.0001.

The hUCMSCs displayed osteogenic potential in the osteogenic medium. On day 7 after osteogenic induction, staining and semi-quantitative analysis of ALP (Figure 1(a-c)) revealed that the osteogenic medium greatly enhanced ALP activity in comparison to the control. To confirm that cells could properly undergo the late osteogenesis process in the osteogenic medium, we performed Alizarin red staining on day 21 to detect extracellular matrix calcification. Alizarin red staining and semi-quantitative analysis (Figure 1(d-f)) showed that calcium nodule deposits were largely distributed in the osteogenic induced group, while hardly found in the control cultures. The qRT-PCR revealed that the expression levels of ALP, Runx2, and OPN of hUCMSCs were significantly increased after osteogenic induction on day 7 (Figure 1(g-i)). All of these results indicated that hUCMSCs had differentiated into osteogenic cells induced by the osteogenic medium.

### Expression of differentially expressed lncRNAs

The profiles of lncRNAs in three cultures that had been osteogenically induced were compared to profiles in three control cultures. Sequencing of RNA from the three induced cultures generated 92289676, 91596000, and 99497528 clean reads, while sequencing from the control cultures generated 74,761,478, 85884244, and 93375536 clean reads (Table 3). In total, 68925 and 28361 unique lncRNAs were identified, respectively, in the induced or control cultures.

**Table 3.** Summary of data from lncRNA-seq for the osteogenic induced groups and non-osteogenic induced groups

Sample Name	Raw reads fold change	Clean reads	Aligned reads	Unique lncRNAs
Induced1	92,321,904	92,289,676	60,746,205	68925
Induced2	91,673,540	91,596,000	54,778,213	
Induced3	99,582,072	99,497,528	43,040,724	
Control1	74,813,166	74,761,478	62,836,053	28361
Control2	85,919,716	85,884,244	60,097,742	
Control3	93,435,824	93,375,536	52,452,845	

**Figure 2.**
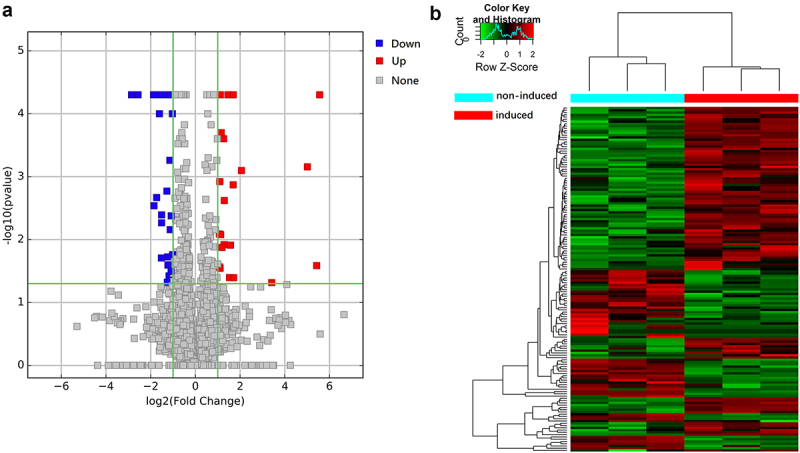
Volcano plot and heat map of differentially expressed lncRNAs. (a) Volcano plot of lncRNAs between hUCMSCs induced to undergo osteogenic differentiation (‘induced’) or not (‘non-induced’). Each point represents a lncRNA. (b) Clustered heatmap of lncRNAs differentially expressed between induced and non-induced cultures. Each column indicates one sample; each row, one lncRNA. The color from blue to red represents increasing expression from low to high. Values are the Z-scores of log_2_ (FPKM+1), where FPKM refers to fragments per kilobase of exon per million fragments mapped.

After defining differentially expressed lncRNAs as those showing at least a 2-fold change between the two culture conditions (*P* < 0.05), we identified 343 differentially expressed lncRNAs, of which 115 were upregulated and 228 were downregulated upon osteogenic induction (Figure 2). The top 20 up- and downregulated transcripts are described in Table 4.

**Table 4.** Details of the top 20 lncRNAs upregulated or downregulated during osteogenic differentiation of hUCMSCs

Upregulated			
Transcript_id	Gene_id	Fold change	p_value
ENST00000414790	ENSG00000130600	84.038004	0.00005
uc021qbx.2	AK311497	56.460109	0.0007
TCONS_00005314	XLOC_002461	5.8994072	0.02585
ENST00000418001	ENSG00000236209	5.5513552	0.0415
ENST00000426962	ENSG00000237940	5.5510858	0.04025
ENST00000606162	ENSG00000271738	5.3441111	0.00005
ENST00000577988	ENSG00000265185	4.5005771	0.0008
ENST00000534671	ENSG00000246273	4.185268	0.0123
ENST00000429456	ENSG00000237940	4.117932	0.00005
ENST00000602478	ENSG00000270022	4.0364863	0.00135
ENST00000571722	ENSG00000262074	3.8309442	0.00005
ENST00000430555	ENSG00000233806	3.8303069	0.00815
uc003yud.3	WISP1	3.4901039	0.00005
ENST00000589987	ENSG00000261040	3.4118637	0.01335
ENST00000451070	ENSG00000237940	3.4105159	0.0401
uc001lvf.3	INS-IGF2	3.3844467	0.00005
ENST00000560743	ENSG00000259450	3.3411622	0.0007
ENST00000540811	ENSG00000255864	3.0725788	0.00025
ENST00000426161	ENSG00000198468	3.0506782	0.0366
ENST00000602957	ENSG00000269927	3.0294586	0.00005
**Downregulated**			
Transcript_id	Gene_id	Fold change	p_value
uc021zbs.1	EEF1A1	−57179711	0.00215
uc002idf.3	NBR2	−20.12588	0.0054
ENST00000587182	ENSG00000272975	−16.69912	0.0001
TCONS_00016279	XLOC_007654	−14.77883	0.0376
ENST00000423943	ENSG00000224259	−10.37457	0.00005
ENST00000605886	ENSG00000272405	−9.696271	0.00205
NR_027405	MTHFD2	−8.154916	0.00005
uc001jet.3	AGAP9_2	−7.727169	0.03855
NR_130154	FARSB	−7.36145	0.04605
ENST00000602597	ENSG00000269947	−7.346668	0.0497
ENST00000433753	ENSG00000012171	−7.003131	0.02515
TCONS_00027719	XLOC_013282	−6.955672	0.00005
ENST00000533920	ENSG00000247095	−6.732069	0.0255
ENST00000418368	ENSG00000215190	−6.399681	0.03775
ENST00000561521	ENSG00000261824	−5.520887	0.00055
ENST00000607314	ENSG00000272327	−5.349522	0.00175
ENST00000569291	ENSG00000261474	−5.034226	0.032
ENST00000324446	ENSG00000176728	−4.407498	0.049
TCONS_00015147	XLOC_007190	−4.376325	0.0196
ENST00000609207	ENSG00000223764	−4.338451	0.00005

### Validation of RNA sequencing

To validate the accuracy of the sequencing, we randomly selected five up- and five downregulated lncRNAs and analyzed their expression using qRT-PCR. This assay confirmed that ENST00000414790, ENST00000577988, ENST00000580476, ENST00000584923, and ENST00000363359 were upregulated, while NR_027405, ENST00000428008, ENST00000448718, ENST00000483140, and NR_109779 were downregulated (Table 5). This agreement suggests that our RNA sequencing is reliable.

**Table 5.** Comparison of results from qRT-PCR and RNA sequencing

	qRT-PCR			RNA sequencing	
Gene	Fold change	p_value	Regulation	Fold change	p_value
ENST00000414790	132.3	<0.0001	up	84.038004	0.00005
ENST00000577988	1.432	0.0475	up	4.5005771	0.0008
ENST00000580476	1.97	0.0188	up	2.7522662	0.00005
ENST00000584923	2.349	0.0156	up	2.1880565	0.0287
ENST00000363359	1.278	0.0064	up	2.0832269	0.00005
NR_027405	−1.772	0.0045	down	−8.154916	0.00005
ENST00000428008	−1.243	0.0065	down	−3.774093	0.0029
ENST00000448718	−1.431	0.0347	down	−2.674149	0.00005
ENST00000483140	−1.266	0.0073	down	−2.463774	0.0017
NR_109779	−1.524	0.002	down	−2.161331	0.00005

### Functional enrichment of differentially expressed lncRNAs

**Figure 3.**
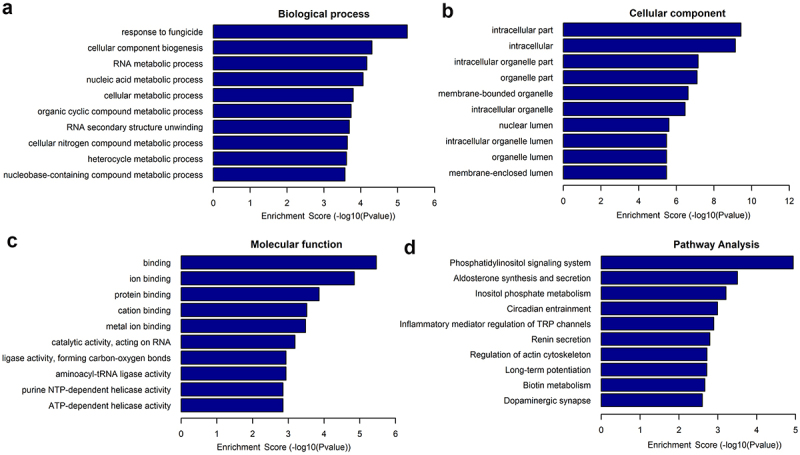
Enrichment of differentially expressed lncRNAs in GO terms and KEGG pathways. GO terms: (a) Biological processes, (b) cellular components, and (c) molecular functions. (d) KEGG pathways.

The potential roles of differentially expressed lncRNAs in the osteogenic differentiation of hUCMSCs were explored using GO terms and KEGG pathways. A higher enrichment score [-log(P-value), *P*< 0.05] for a given term or pathway indicates a more significant correlation. The potential functions of the differentially expressed lncRNAs with the 10 highest enrichment scores are shown in Figure 3(a-c). Upregulated lncRNAs were most significantly associated with the GO biological process ‘response to fungicide’, ‘cellular component biogenesis’, and ‘RNA metabolic process’; with the GO cellular components ‘intracellular compartment’, ‘intracellular’, and ‘intracellular organelle compartment’; and with the GO molecular functions ‘binding’, ‘ion binding’, and ‘protein binding’. We identified 49 KEGG pathways that were significantly related to differentially expressed lncRNAs, of which 38 pathways were upregulated and 11 were downregulated after osteogenic induction. The upregulated pathways with the 10 highest enrichment scores are shown in Figure 3(d). The KEGG pathways most significantly associated with lncRNAs were ‘phosphatidylinositol signaling system’, ‘aldosterone synthesis and secretion’, and ‘inositol phosphate metabolism’.

### Construction of the ceRNA network of interacting lncRNAs, miRNAs, and mRNAs

**Figure 4.**
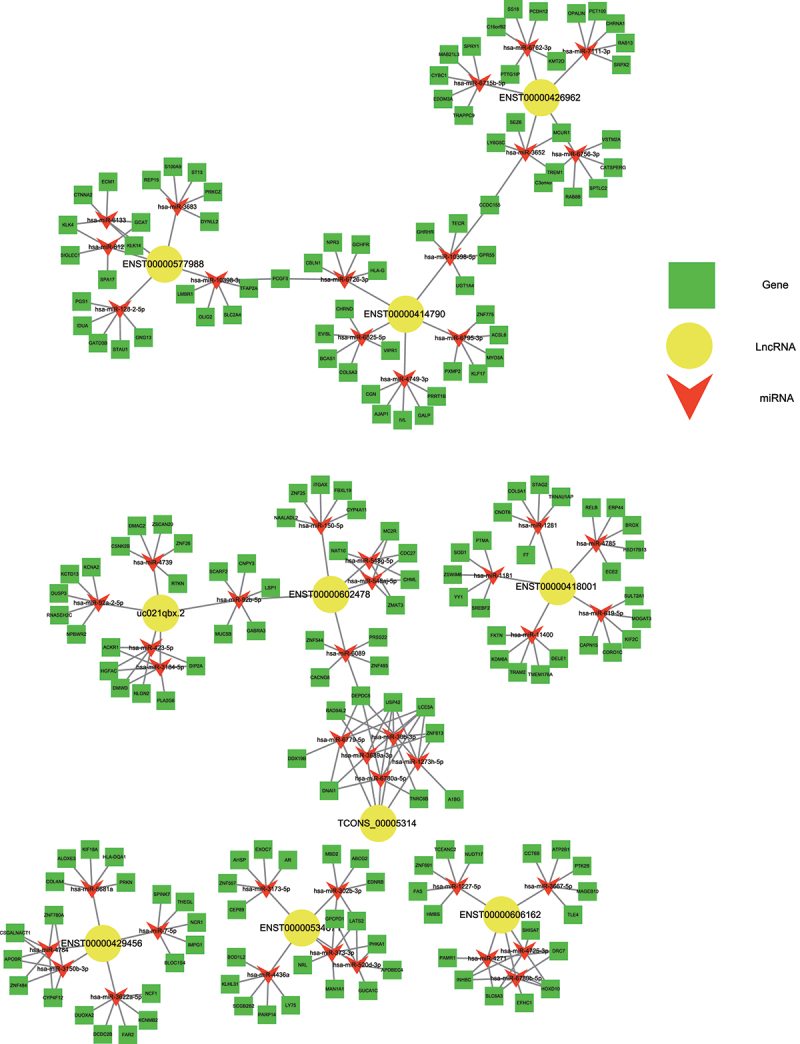
Construction of the ceRNA network describing lncRNA-miRNA-mRNA interactions during osteogenic differentiation of hUCMSCs.

lncRNAs can bind to miRNAs and thereby prevent the latter from binding to their target mRNA and inhibiting its translation. To predict the lncRNA-miRNA-mRNA interactions of the differentially expressed lncRNAs, lncRNA-miRNA-mRNA-associated ceRNA networks were constructed. We constructed six ceRNA networks based on the top 10 upregulated lncRNAs (Table 6): ENST00000414790, uc021qbx.2, TCONS_00005314, ENST00000418001, ENST00000426962, ENST00000606162, ENST00000577988, ENST00000534671, ENST00000429456, and ENST00000602478 (Figure 4). Among these lncRNAs, ENST00000414790 and uc021qbx.2 differed between induced and control cultures severalfold more than the eight other lncRNAs did. The Ensembl database indicated that for both of these lncRNAs, the full-length transcript was lncRNA H19, with ENST00000414790 accounting for the largest number of transcripts from H19. Thus, we chose lncRNA H19 to explore its potential regulatory role in the osteogenic differentiation of hUCMSCs.

**Table 6.** Filtered differentially expressed lncRNAs

Transcript_id	Gene_id	Fold change	p_value	Regulation	lncRNA_source
ENST00000414790	ENSG00000130600	84.04	0.00005	up	Ensembl
uc021qbx.2	AK311497	56.46	0.0007	up	UCSC_knowngene
TCONS_00005314	XLOC_002461	5.9	0.02585	up	TCONS
ENST00000418001	ENSG00000236209	5.55	0.0415	up	Ensembl
ENST00000426962	ENSG00000237940	5.55	0.04025	up	Ensembl
ENST00000606162	ENSG00000271738	5.34	0.00005	up	Ensembl
ENST00000577988	ENSG00000265185	4.5	0.0008	up	Ensembl
ENST00000534671	ENSG00000246273	4.19	0.0123	up	Ensembl
ENST00000429456	ENSG00000237940	4.12	0.00005	up	Ensembl
ENST00000602478	ENSG00000270022	4.04	0.00135	up	Ensembl

### Overexpression of lncRNA H19 enhances osteogenic differentiation of hUCMSCs

**Figure 5.**
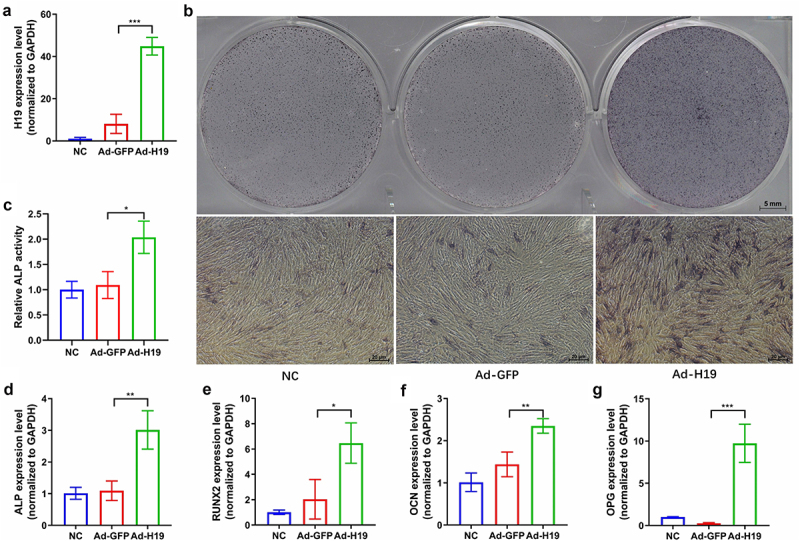
Overexpression of the lncRNA H19 enhances osteogenic differentiation of hUCMSCs. Cultures of hUCMSCs were uninfected (nonspecific control, ‘NC’) or infected with recombinant adenovirus expressing GFP (‘Ad-GFP’) or lncRNA H19 (‘Ad-H19’). (a) qRT-PCR results from day 3 confirmed the overexpression of lncRNA H19. (b) ALP staining on day 7. (c) Semi-quantitative analysis of ALP activity on day 7. (d-g) Expression of osteogenic markers ALP, RUNX2, OCN, and OPG after osteogenic induction on day 7. *P < 0.05, **P < 0.01, ***P < 0.001.

Our RNA sequencing and qRT-PCR results showed upregulation of the lncRNA H19 at seven days after osteogenic differentiation of hUCMSCs (Table 5). To confirm the effect of lncRNA H19 on osteogenesis, we overexpressed lncRNA H19 in hUCMSCs using recombinant adenovirus. Overexpression of lncRNA H19 was confirmed by qRT-PCR on day 3 after infection (Figure 5(a)). On day 7 after infection, ALP staining and semi-quantitative analysis revealed that ALP activity in lncRNA H19 overexpressed hUCMSCs was greatly enhanced in comparison to the hUCMSCs infected with control adenovirus (Figure 5(b,c)). The qRT-PCR results from day 7 indicated that lncRNA H19 overexpressed hUCMSCs had a significantly higher expression of the osteogenic markers ALP, RUNX2, OCN, and OPG than hUCMSCs infected with control adenovirus (Figure 5(d-g)). These results suggest that the lncRNA H19 helps drive osteogenic differentiation of hUCMSCs.

## Discussion

Regeneration of bone defects caused by tumor resection, infection, and trauma is a clinical challenge for orthopedic surgeons [46]. To treat these defects, bone grafting materials including autologous bone grafts (autografts), allogenic bone grafts (allografts), and synthetic grafts have been extensively investigated [1]. Autografts are considered the gold standard to treat bone defects because of their osteoconduction and osteoinduction. However, autografting suffers from several major disadvantages including donor site morbidity and limited bone supply [47]. Allografts and synthetic grafts can avoid the drawbacks of autografts. However, allografts have several disadvantages of their own, including bacterial infection and disease transmission, while synthetic grafts integrate poorly with host bone and are susceptible to wear and tear [48,49].

To find alternative therapies to treat bone defects, cell-based tissue engineering using scaffolds seeded with cells to promote bone regeneration has been suggested [2]. BMSCs can easily be harvested from bone marrow and are regarded as the ‘gold standard’ among MSCs [50]. Therefore, BMSCs are widely used in bone tissue engineering and cell-based therapies as cytokine pumps and replacement cells [5]. However, BMSCs must be harvested through an invasive procedure, and relatively few cells can be recovered from each patient [4]. Moreover, their slow proliferation means that several weeks are needed to expand them *in vitro* before clinical use [5]. In addition, BMSCs from older and diseased individuals show lower quantity and quality [51]. Therefore, alternative MSC sources are needed for tissue engineering.

hUCMSCs have been suggested as an excellent alternative source of MSCs for bone regeneration [7]. Unlike BMSCs, hUCMSCs can be collected noninvasively, proliferate rapidly, and show higher differentiation capability [^8–10^]. Whereas BMSCs show notably longer doubling time after the sixth passage [52], hUCMSCs maintain a steady doubling time until the tenth passage. hUCMSCs are also less immunogenic than hBMSCs because they do not express costimulatory ligands including CD86, CD80, or CD40, they do not express major histocompatibility complex (MHC) class II molecules, and they express only low levels of MHC class I molecules [53]. All these advantages render hUCMSCs attractive for cell-based bone tissue engineering.

Before hUCMSCs can be exploited for regenerative bone therapy, the regulation of their osteogenic differentiation needs to be understood. Physical, chemical, and biological signals can influence MSC differentiation via a batch of signaling pathways, which ultimately trigger regulatory cascades at both the transcriptional and post-transcriptional levels [12,54]. It has been reported that many critical signaling pathways help regulate MSC differentiation, such as pathways involving Hedgehog, Notch, Wnt, and TGF-β/BMP [12]. And lncRNAs may help regulate these pathways, since lncRNAs have been shown to exert crucial roles in many biological and pathological processes, including metabolism, cellular development, tumorigenesis, immune response, and genetic imprinting [55]. Besides, it has been demonstrated that lncRNAs regulate the differentiation of MSCs [13], including their osteogenic differentiation [20^,23–28^]. In recent years, the expression profiles and functions of lncRNAs in the osteogenic differentiation of hBMSCs have been investigated [23,40,56,57]. However, the role of lncRNAs in hUCMSC osteogenic differentiation remains largely unknown.

The present study first revealed the expression profiles of lncRNAs during the osteogenic differentiation of hUCMSCs and further analyzed these lncRNAs using bioinformatic analyses. We identified 343 lncRNAs differentially expressed during osteogenic differentiation, of which 115 were upregulated and 228 were downregulated. To validate the accuracy of the sequencing-based results, 10 differentially expressed lncRNAs were analyzed by qRT-PCR. The qRT-PCR results obtained from these 10 lncRNAs were consistent with the sequencing results, demonstrating that the sequencing results were reliable. The potential functions of differentially expressed lncRNAs were explored by searching for enrichment in GO terms and KEGG pathways. GO analysis showed that the main GO terms were found to be associated with the cellular component, such as intracellular part, intracellular, intracellular organelle part, and organelle part. The KEGG pathways analysis indicated that many pathways such as ‘phosphatidylinositol signaling system’, ‘aldosterone synthesis and secretion’, and ‘inositol phosphate metabolism’ may be involved in the osteogenic differentiation of hUCMSCs. Among these pathways, the phosphatidylinositol signaling system is closely related to bone metabolism. It has been reported that activation of phosphatidylinositol 3-kinase (PI3K)/Akt signaling pathway could mediate osteogenic differentiation of MSCs [58]. Besides, the majority of the phosphatidylinositol family including PI3K plays a regulatory role in the osteogenesis of MSCs by the regulation of BMP-2 gene expression via mitogen-activated protein kinases signaling pathway [59,60].

To predict some core regulating factors in the osteogenic differentiation of hUCMSCs, we then chose the top 10 upregulated lncRNAs to construct potential interactions between lncRNA, miRNAs, and mRNAs by ceRNA networks. In the present study, we found the full-length transcript of the top 2 upregulated lncRNAs (ENST00000414790 and uc021qbx.2) was lncRNA H19. Thus, we chose lncRNA H19 to explore its potential regulatory role in the osteogenic differentiation of hUCMSCs. Our study showed that lncRNA H19 upregulation resulted in increased ALP activity and higher expression of the osteogenic markers ALP, RUNX2, OCN, and OPG in hUCMSCs, suggesting lncRNA H19 was regulating the osteogenic differentiation of hUCMSCs as an enhancer. The lncRNA H19 is one of the most well-known conserved non-coding transcripts expressed from the maternal allele [61]. It has been demonstrated that lncRNA H19 presents a significant role in mediating the osteogenesis of MSCs [62]. LncRNA H19 was reported to be upregulated in the osteogenic differentiation of hBMSCs in several studies [20,23,63]. However, another study in human adipose-derived stem cells got the reverse trend, which may be explained by the differential tissue- and cell-specific expression manner of lncRNA H19 during embryogenesis [64]. Besides, Huang et al reported that lncRNA H19 promoted osteogenesis of hBMSCs via the TGF-β1/Smad3/HDAC pathway, and miR-675 partially mediated this pro-osteogenic function [20]. Meanwhile, Liang et al demonstrated that lncRNA H19 functioned as a ceRNA for miR-141 and miR-22 to direct potentiate the Wnt/β-catenin pathway, leading to the enhancement of osteogenesis of hBMSCs [63]. Moreover, lncRNA H19 could also up-regulate focal adhesion kinase by serving as a ceRNA for miR-138 to promote tension-induced osteogenic differentiation of hBMSCs [65]. In addition, an *in vitro* study of mice reported that lncRNA H19 mediated the expression level of ligand-dependent corepressor by acting as a ceRNA for miR-188, thus regulating the balance between osteogenic and adipogenic differentiation of BMSCs [21]. Overall, these studies demonstrated that the lncRNA H19-mediated lncRNA-miRNA-mRNA regulatory axis plays an important role in mediating the osteogenesis of MSCs. Therefore, the underlying ceRNA mechanisms of lncRNA H19 in regulating the osteogenesis of hUCMSCs are needed to be further clarified.

Nevertheless, our findings should be treated with caution in light of several limitations. First, we profiled lncRNA expression and observed the expression of gene markers during early osteogenic differentiation; such profiling and observation should also be performed at a late stage of the osteogenesis process. Second, we applied only one approach when constructing the ceRNA network of lncRNA-miRNA-mRNA interactions; using multiple approaches may provide a more accurate result. Third, our sample was small, so our results should be verified and extended in studies with more samples.

## Conclusion

This appears to be the first report of lncRNA expression profiles during the osteogenic differentiation of hUCMSCs. We explored potential functions of differentially expressed lncRNAs based on enrichment in GO terms and KEGG pathways. We also predicted the ceRNA network of interactions among lncRNAs, miRNAs, and mRNAs. In particular, we identified the lncRNA H19 as a potential driver of osteogenic differentiation of hUCMSCs. These findings provide numerous testable hypotheses to guide experiments to elucidate how lncRNAs, miRNAs, and mRNAs regulate the osteogenic differentiation of hUCMSCs.

## Data Availability

Data is available at NCBI Sequence Read Archive (SRA, https://www.ncbi.nlm.nih.gov/sra) database, accession numbers: SRX10504596, SRX10504598, SRX10504600, SRX10504602, SRX10504604, SRX10504606.
